# Nutrition and Gut Health: Preparation and Efficacy of Resistant Starch

**DOI:** 10.3390/foods14030471

**Published:** 2025-02-01

**Authors:** Yulong Niu, Li Wang, Huiyi Gong, Shuqing Jia, Qing Guan, Linling Li, Hua Cheng

**Affiliations:** 1 School of Modern Industry for Selenium Science and Engineering, Wuhan Polytechnic University, Wuhan 430048, China; 15221666406@163.com (Y.N.); 15717341028@163.com (L.W.); ghy13774048819@163.com (H.G.); sqing0222@163.com (S.J.); gq1323645363@163.com (Q.G.); 2 National R&D Center for Se-Rich Agricultural Products Processing, Wuhan Polytechnic University, Wuhan 430023, China

**Keywords:** resistant starch, structure, preparation, function, small intestine

## Abstract

Resistant starch (RS) refers to starch varieties that resist digestion by human digestive enzymes. Owing to its distinctive physicochemical attributes and functional capabilities, RS has gained a wide range of applications as a dietary fiber and prebiotic. In terms of structure and functions, RS can be categorized into five distinct types: RS1 through RS5. These types offer dietary benefits, contributing to improved colonic health, the modulation of microbial communities, the reduction in gallstone formation, the enhancement of mineral absorption, and alterations in fat oxidation potential. From a technical standpoint, RS can be manufactured through an array of physical, enzymatic, and chemical modifications. This paper presents a comprehensive review of the existing literature, summarizing the classification, structural features, raw material origins, preparation methodologies, and functionalities of RS. Furthermore, new production technologies and applications of RS, such as 3D printing, provide valuable insights.

## 1. Introduction

Starch constitutes the primary nutritional component in human diets, derived from the fruits, seeds, and roots of various crops, including potatoes, rice, wheat, corn, and cassava [1]. Initially, starch undergoes initial breakdown by salivary amylase secreted in the oral cavity before transitioning through the stomach into the intestine. Here, it is further digested by α-amylase produced by the pancreas [2]. Research has demonstrated that certain starches exhibit resistance to hydrolysis by amylase, rendering them non-absorbable in the digestive tract. Englyst proposed a classification system for starches based on their digestibility. Depending on the speed and extent of digestion, starches can be categorized into three distinct groups: rapidly digestible starch (RDS), which is swiftly digested and absorbed into the bloodstream by enzymes within 20 min during in vivo digestion; slowly digestible starch (SDS), which can be fully digested within a timeframe of 20 to 120 min; and a third category comprising starch that remains undigested and unabsorbed by the small intestine within 120 min. Instead, this starch undergoes fermentation and utilization by intestinal microorganisms upon reaching the large intestine. Englyst and his colleagues were the first to coin the term “resistant starch” (RS) for this type of starch [3,4].

As a natural ingredient, RS holds vast potential for application in both the food and healthcare industries. In addition to the naturally occurring RS discovered by Englyst, various types of RS have been synthesized through the modification of starch. These newly synthesized RSs exhibit superior properties compared to natural RS [5]. RS enhances the crispiness and volume of food, thereby improving overall product quality. Due to its low water-holding capacity and favorable texture, RS is extensively utilized in food processing to augment sensory attributes [6]. Furthermore, as RS remains undigested in the small intestine, it presents promising opportunities for drug encapsulation, enabling the targeted release of drugs at specific bodily sites. As an additive, RS can effectively manage weight and prevent elevations in blood glucose while offering additional physiological benefits [7]. Additionally, RS can be harnessed to develop foods tailored for specific medical purposes. For example, RS exhibits certain therapeutic effects in the prevention and treatment of chronic diseases such as diabetes, obesity, and hyperlipidemia [8]. The substantial demand for RS across diverse fields has fueled research endeavors aimed at synthesizing RS in large quantities to meet the burgeoning market demands.

## 2. RS Structure and Characteristics

### 2.1. Classification of RS

When Englyst and his colleagues initially introduced the concept of RS, they categorized it into three types: RS1, RS2, and RS3. Subsequent to their in-depth research on RS, Englyst and his team discovered a novel type of chemically modified starch, named RS4 [4]. The resistance of RS4 to digestive enzymes is attributed to the chemical alteration of its original functional groups or the incorporation of novel functional groups. This modification leads to the formation of carboxymethyl starch, starch ethers, starch esters, and cross-linked starches [9]. Furthermore, the branches of amylose or amylopectin can interact with lipids to produce starch–lipid complexes, which are impervious to both water and amylase. This particular type of starch has been termed RS5 [10]. The characteristics of different resistant starches are shown in Table 1.

### 2.2. RS1

RS1 denotes physically indigestible starch that is encapsulated within either whole or partially milled grains or seeds [11]. The distinctive structure of RS1 plays a crucial role in mitigating starch hydrolysis, glucose absorption, and maintaining blood glucose equilibrium. By delaying enzymatic breakdown and prolonging glucose release, RS1 is used to limit the prevalence of certain diseases [11]. As a natural starch, the resistance of RS1 to digestion varies according to factors such as particle size, crystalline structure, and density characteristics. Furthermore, the smooth and dense layer on the surface of RS1 starch particles poses a substantial barrier to enzyme–starch interaction, further decreasing starch digestibility [16].

### 2.3. RS2

RS2, another type of natural starch granule, is characterized by a unique compact structure that shields it from digestive enzymes and amylases. Examples of RS2 include raw potato and banana starches [17]. Owing to their compact structure, these starches are not easily digested. However, for the majority of RS2, typical cooking temperatures accompanied by high moisture content often result in starch gelatinization, which disrupts the structure of starch granules and enhances their digestibility [17]. Both RS1 and RS2 undergo slow and incomplete digestion in the small intestine.

### 2.4. RS3

RS3 pertains to starch that acquires resistance to amylase digestion after gelatinization, a result of crystallization during cooling or storage processes. It is also referred to as physically modified starch [3]. Specifically, RS3 is a retrograded starch polymer that forms upon the cooling of gelatinized starch. Owing to its non-digestible properties and physiological functions, such as fostering the proliferation of beneficial intestinal microbial flora and inhibiting the growth of intestinal pathogens, RS3 offers a multitude of health benefits [18]. After the complete gelatinization of starch granules, RS3 is extracted in the form of randomly coiled amylose. Upon cooling, the starch chains rearrange to form a dense left-handed double helix structure, stabilized by hydrogen bonds [19]. This type of RS can be further categorized into RS3a and RS3b, where RS3a represents retrograded amylopectin and RS3b signifies retrograded amylose. Both RS3a and RS3b are efficacious in markedly reducing the release of blood glucose. Chain length distribution is a pivotal characteristic of starch’s primary structure. When the gelatinized starch is cooled, the amylose and amylopectin molecules will rearrange and form an ordered crystal structure, and shorter starch chains are more likely to cluster together to form new crystalline zones while longer chains may remain amorphous. Thus, RS3 formation causes an increase in the relative proportion of short chains and a decrease in long ones in starch [20]. During the in vivo digestion of RS3a and RS3b, the proportion of A chains gradually increases in the digestive residues, while the proportion of B chains decreases correspondingly [21].

### 2.5. RS4

RS4 denotes starch that has undergone chemical modification through processes such as etherification, esterification, or reaction with various chemical compounds, including sodium trimetaphosphate, sodium tripolyphosphate, epichlorohydrin, and phosphoryl chloride [22]. The incorporation of functional groups into the starch chain aids in stabilizing starch pastes and gels, thereby mitigating retrogradation [23]. RS4 exhibits resistance to digestive enzymes due to the chemical modification of its original functional groups or the introduction of novel functional groups. This leads to the formation of compounds like carboxymethyl starch, hydroxypropyl starch ethers, starch phosphates, and cross-linked starches [1]. RS4 starch granules are typically characterized by a rough surface and a hollow structure, encompassing both crystalline and amorphous regions. The surface of these granules is less smooth compared to natural starch but retains a relatively intact particle structure. As the number of chemical groups increases, the intermolecular hydrogen bonds are progressively disrupted [24,25]. Chemical modification can change the degree of crosslinking between starch chains, which affects the chain length ratio. For example, esterification can add fatty acid chains at specific positions to change the original chain length, and these modifications can also prevent the action of digestive enzymes, allowing more long chains to be retained [26].

### 2.6. RS5

RS5 emerges when starch, under the influence of external conditions, undergoes intramolecular hydrogen bond interactions. This leads to the rotation of the chain structure of amylose, resulting in the formation of a V-type crystalline structure with thermodynamic stability [25]. The creation of starch–lipid V-type complexes diminishes starch digestibility [27]. RS5 is regarded as a promising additive for individuals with specific dietary needs, particularly those with metabolic syndromes. It demonstrates a high degree of resistance to digestive enzymes, indicating that it is not readily digested and absorbed in the small intestine of humans. However, upon reaching the large intestine, RS5 can undergo fermentation by intestinal microorganisms, yielding short-chain fatty acids such as butyric acid, which confer numerous health benefits to the human body [28]. In addition to starch–lipid V-type complexes, recent research has delved into the development of other starch V-type complexes, including starch–glycerol, starch–protein, starch–polyphenol, and starch–other polysaccharides. These complexes exhibit similar traits and can thus be categorized under RS5 [29]. RS5 formation depends on the interaction between amylose and lipids, especially since long amylose is more likely to repose with lipids than short amylose. The formation of this complex not only changes the physical properties of starch but may also affect the effective length of the starch chain, where those involved in complex formation are no longer susceptible to digestive enzymes, thus effectively prolonging their presence in the digestive tract [30].

### 2.7. Method for Determining the Structure of Resistant Starch

The structural measurement of RS involves its physical, chemical, and biological properties. To comprehensively understand the nature of RS, various techniques can be employed to analyze its microstructure, crystalline morphology, and molecular composition.

Scanning Electron Microscopy (SEM) is an imaging tool widely used in materials science, biology, medicine, and other fields, particularly for microstructural analysis. By scanning the sample surface point by point using a focused electron beam and collecting secondary electrons or backscattered electrons emitted by the sample, SEM forms an image [31]. In the study of RS, SEM plays a crucial role in providing insights into changes in starch granule structure. For instance, SEM images clearly demonstrate the structural transformation of corn starch from an amorphous region to a crystalline region after no treatment and enzymatic treatment [32].

X-ray diffraction (XRD) technology is also essential in RS research. It is not only used to determine the crystallinity and crystal structure of RS but also reflects the crystalline properties of starch by detecting helical structures [33]. Starch granules consist of both crystalline and amorphous regions internally. The crystalline region, primarily composed of amylopectin molecules, is relatively dense, while the amorphous region, mainly composed of amylose molecules, is susceptible to external forces [34]. Based on the characteristic peaks in the X-ray diffraction pattern, starch crystal structures can be classified into four types: A-type, B-type, C-type, and V-type. The characteristic peaks of A-type starch are at 15°, 17°, 18°, and 23°; those of B-type starch are at 5.6°, 17°, 22°, and 24°; those of C-type starch are at 5.6°, 15°, 17°, 19°, 23°, and 26°; and those of V-type starch are at 7°, 13°, and 19.9° [35]. The crystalline structure of starch is related to food properties. For example, the crystallinity at the characteristic peak of 15° is significantly positively correlated with short chains of amylose (DP13-24), as well as with gelatinization end temperature and water solubility. The crystallinity at the characteristic peaks of 17–18° is significantly positively correlated with the gelatinization onset temperature and water solubility. The crystallinity at the diffraction peak of 23° is significantly positively correlated with the gelatinization end temperature and gelatinization peak time [36].

In research, 13C CP/MAS NMR (Nuclear Magnetic Resonance) spectroscopy can be utilized to measure the molecular structure, relative crystallinity, and double helix content of RS. Relative crystallinity is a concept used to describe the proportion of crystalline regions within a material. Double helix content has a significant impact on the formation of RS [37]. For instance, lotus seed starch with high double helix content exhibits higher RS content [38].

Fourier Transform Infrared (FTIR) spectroscopy reveals the internal molecular structure of RS by detecting the presence and vibration modes of specific functional groups. The ratio of 1047 cm^−1^ to 1022 cm^−1^ (R1047/1022) is particularly important in assessing changes in RS structural content. Additionally, FTIR can measure changes in hydrogen bonds during starch processing, as these changes affect starch retrogradation [39]. For example, three different methods were used to treat purple sweet potato starch, and the results, measured using XRD and FTIR, indicated that all three treatments converted the crystal structure of purple sweet potato starch from C-type to B-type, without generating new groups during the modification process [40].

High-Performance Liquid Chromatography (HPLC) can quantify RS by systematically identifying and separating carbohydrate components, potentially providing more accurate RS content measurements [41]. Gel Permeation Chromatography (GPC) is used to determine the molecular weight of starch, which influences the physicochemical properties of RS, such as solubility and viscosity [42].

Starch chain length refers to the distribution of the length and number of α-1,4-glucose chains and α-1,6-glucose chains in starch molecules. The length distribution of starch chains affects the rheological properties, digestion resistance, and application of starch in food processing [43]. Chain length distribution is an important feature of starch’s primary structure, primarily including A-chains (DP6-12), B1-chains (DP13-24), B2-chains (DP25-36), and B3-chains (DP > 36). GPC and Ion Chromatography (IC) are commonly used methods to measure starch chain length. GPC is simple to operate and has a wide detection range but cannot fully distinguish glucose chains of different degrees of polymerization. IC determines the chain length distribution of amylopectin by judging the distribution of various glucose chains based on the peak area and number of chromatographic peaks. Studies have found that the amylose content and amylose chain length distribution significantly affect starch digestibility and gelatinization properties. The texture of foods with high glutinous rice content can be improved by altering the low ratio of long and short branches of amylopectin. Changes in chain lengths are closely related to food properties and digestion characteristics [44]. For example, short amylopectin chains are negatively correlated with hardness but positively correlated with adhesiveness and cohesion [45].

## 3. Preparation Methods of RS

**Figure 1:**
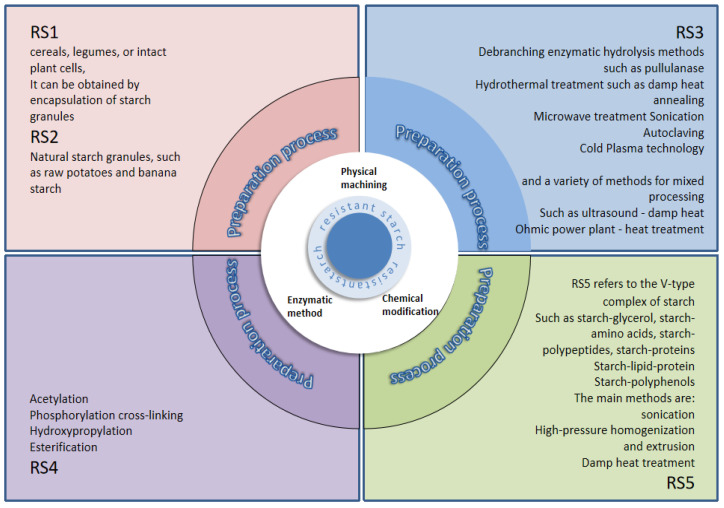
Methods for making different types of resistant starch.

The preparation method is a crucial factor influencing the yield of RS, which can be categorized into physical, chemical, and enzymatic methods based on their principles. The preparation method and yield of resistant starch are shown in Table 2 [46]. Physical methods are advantageous in terms of low cost, environmental friendliness, and safety. They primarily include two aqueous heat treatment processes (heat-moisture treatment and annealing treatment) and various non-aqueous heat treatment processes (autoclaving, ultrasonic treatment, microwave treatment, high-pressure homogenization, etc.) [47]. Chemical methods mainly involve acid hydrolysis, crosslinking treatment, esterification, acetylation, etc., introducing new functional groups through chemical modification to alter the original physicochemical properties of starch. The different types of resistant starch are shown in Figure 1 [48].

### 3.1. Physical Methods

Heat–moisture treatment (HMT) refers to the process of heat-treating starch at a temperature above the gelatinization temperature, with limited moisture content (typically 10–30%) for a period of time (usually 15 min to 16 h) [69]. Due to its simple process and ease of management, HMT is relatively easy to implement in industrial production. Studies have found that HMT can induce changes in the particle surface, degree of swelling, amylose content, crystallinity, and gelatinization parameters of starch and a series of starch structures, resulting in changes in food properties. These changes vary with the moisture content during treatment and the source of starch [70]. In a study on the effect of damp heat treatment on the structure and digestibility of sweet potato starch, the relative crystallinity and short-range order of sweet potato starch decreased, the starch molecules rearranged, the surface of starch granules appeared depressed, and the content of RS increased [71].

Annealing treatment, also known as tempering treatment, involves heat treatment at a temperature above the glass transition temperature but below the gelatinization temperature [72]. During annealing, changes in molecular structure occur in the amorphous region of starch granules [73]. The limited but reversible swelling of starch granules allows them to move within the crystalline regions, thereby altering the physicochemical properties and structure of starch [74]. This process facilitates rearrangement within starch granules without causing gelatinization. It has minimal impact on starch granule structure and is suitable for production requiring the maintenance of starch granule integrity, holding broad promise in food processing [75].

Ultrasonic treatment can alter both the amorphous and crystalline regions of starch, thereby changing the RS content and functional properties of different starches [76]. Ultrasonic treatment is generally used in conjunction with other methods. For instance, ultrasonic-assisted enzymatic hydrolysis can significantly increase the enzymatic hydrolysis rate and amylose content. Compared to traditional enzymatic hydrolysis, RS subjected to ultrasonic treatment exhibits higher solubility, larger particle size, higher crystallinity, a stable double helix structure, and higher surface roughness [77]. For example, the yield of RS prepared using a combined hydrothermal–alkali–ultrasonic method is higher than that obtained by a hydrothermal method or hydrothermal–alkali method alone [78].

**Figure 2:**
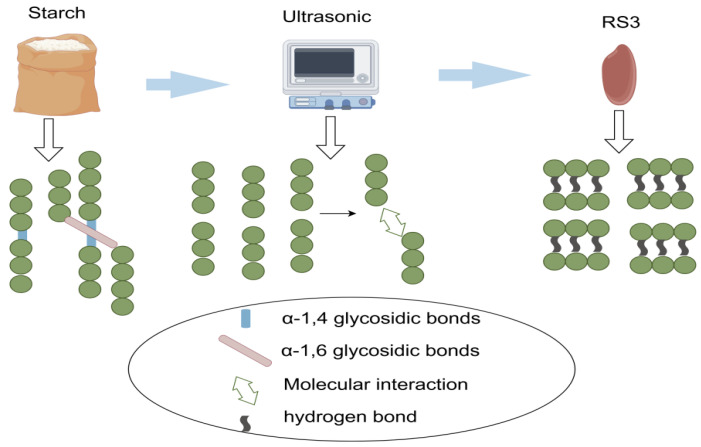
Preparation of RS3 using the microwave method. After microwave treatment, the α-1,4-glycosidic bonds in starch are broken and then recombined through intermolecular interactions to form starch structures connected by hydrogen bonds.

Microwave radiation is a method that utilizes microwave energy for the rapid heat treatment of starch, influencing its molecular structure, physicochemical properties, and digestion characteristics. This is shown in Figure 2 [79]. By mixing starch with water and placing it in a microwave field, the starch rapidly heats up under microwave action. The advantages of this method include fast heating speeds, short treatment times, and uniform heating as microwave energy can penetrate the interior of materials [80]. Microwave treatment, particularly at high power levels (8 and 10 W/g), can produce a higher degree of order and amorphous structures, resulting in high RS content and lower digestibility [81].

High-pressure homogenization (HPH) involves the application of high pressure in a very short time, causing shear forces and temperature increases due to the applied pressure [82]. HPH treatment of starch results in partial gelatinization, with the degree of gelatinization increasing as the homogenization pressure increases, leading to changes in starch particle size [83]. For instance, HPH treatment reduces the particle size of high-amylose corn starch. At 250 MPa, the starch particles are destroyed, and the amylopectin structure changes, while the high-amylose corn starch structure remains unchanged as B-type starch [84].

### 3.2. Chemical Methods

Acid hydrolysis is one of the widely used modification methods for producing resistant starch. It can alter the structure of amylopectin molecules, amylose content, chain length distribution, and the morphology of starch granules [85]. For instance, starch granules remain relatively intact during the first four days of acid hydrolysis. However, severe corrosion is observed after the fifth day, leading to significant damage to the starch granules. By the seventh day, the starch granules are completely fragmented into small pieces [86]. In another example, as the acid hydrolysis time increases, the complexation index of starch–myristic acid complexes prepared through acid hydrolysis decreases significantly, indicating that acid hydrolysis hinders the formation of starch–MA complexes [87].

Esterification, which involves incorporating ester groups into non-esterified molecules, is one of the most widely used chemical modification methods to improve the physicochemical properties of natural starch [88]. Citric acid is commonly used as a safe food additive and as an acidifying and esterifying agent in the pharmaceutical and food industries [89]. Higher RS content is observed in esterified starches with high amylose content (≥50%) [90]. Phenolic acids also serve as effective esterifying agents. Phenolic acid-esterified starch contains more RS than natural starch and has a lower glycemic index [91].

Phosphorylation cross-linking is a chemical modification technique used to improve the structural properties of starch. It can alter the gelatinization characteristics of starch, making starch pastes stable under high acidity and high shear conditions [92]. Cross-linking agents such as sodium trimetaphosphate and sodium tripolyphosphate react with the hydroxyl groups in starch molecules to form a cross-linked structure, enhancing the heat resistance and enzyme resistance of starch [93].

Acetylation involves replacing hydroxyl groups in starch chains with acetyl groups, thereby altering the molecular structure and properties of starch [94]. For instance, acetylated noodle starch exhibits better transparency, condensed volume ratio, hydrophilicity, and lipophilicity compared to natural noodle starch. These properties increase with the degree of substitution. Acetylated noodle starch reduces the content of RDS and increases the content of slowly digestible starch and RS. Additionally, the color, texture, and tensile properties of acetylated noodle starch are also improved [95].

### 3.3. Enzymatic Method

The primary principle of preparing RS through the enzymatic method involves using specific enzymes (such as pullulanase and thermostable α-amylase) to hydrolyze starch, thereby improving the starch chain length and crystallinity. The enzymatic method is a clean technology that provides more environmentally friendly and consumer-safe solutions for starch modification [96]. These starch-modifying enzymes can be classified into four categories based on their mode of action on starch molecules: exoenzymes, endoenzymes, transferases, and debranching enzymes.

Exoenzymes, such as glucoamylase, progressively cleave α-1,4 glycosidic bonds from the non-reducing end in units of maltose. The reaction stops when an α-1,6 glycosidic branch point is encountered, producing limit dextrins with relatively high molecular weights. Endoenzymes, such as α-amylase, can randomly cleave α-1,4 glycosidic bonds within starch molecules. However, α-amylase alone cannot completely hydrolyze starch into glucose.

Transferases are enzymes that transfer glycosyl groups between starch molecules. For example, maltase can hydrolyze α-1,4 glycosidic bonds in starch chains and transfer the cleaved glucose molecules to α-1,6 glycosidic bonds. Debranching enzymes are specifically responsible for hydrolyzing α-1,6 glycosidic bonds at branch points in starch. For instance, pullulanase hydrolyzes α-1,6 glycosidic bonds on the branch side of amylopectin, producing maltotriose and maltooligosaccharides [97,98].

The main limitation of using enzymatic methods is that natural starch granules are dense semicrystalline materials with high resistance to enzyme penetration and hydrolysis, making them slowly hydrolyzed by digestive enzymes. Therefore, the structure of natural starch granules must be disrupted to enhance the efficiency of hydrolytic enzymes [99]. For example, oat RS prepared via ultrasonic-assisted enzymatic hydrolysis has a B + C-type crystal structure, with higher RS content, larger particle size, and higher relative crystallinity compared to autoclaved RS [100].

### 3.4. 3D Printing Technology

Three-dimensional printing technology is a technology that builds objects layer by layer based on computer-aided design models. In the food industry, where 3D printing is already being used to create food products with complex geometries, for resistant starch, 3D printing offers a new way to control its structure and digestive properties to develop healthier foods. In the process of 3D printing, through hot extrusion under specific conditions, part of the amylose is recrystallized to form a resistant form that is difficult to digest and absorb by the human body [101].

**Figure 3:**
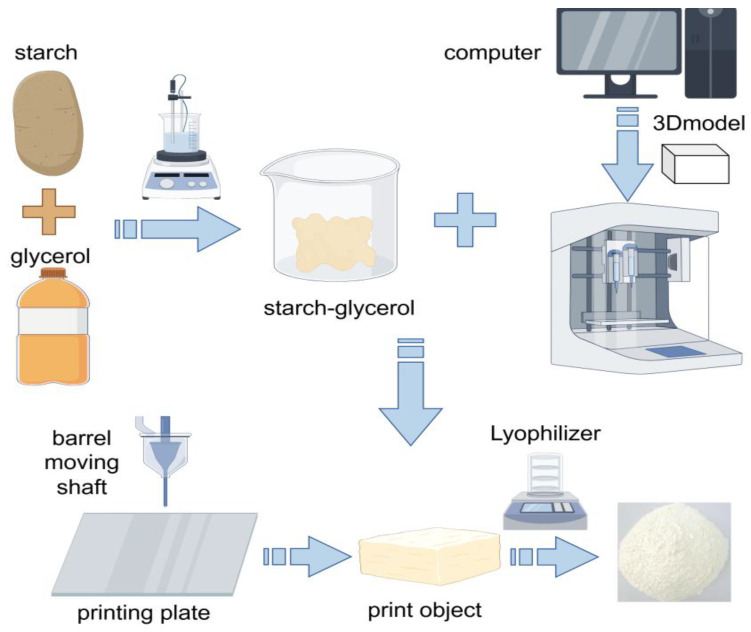
Three-dimensional printing preparation of RS. Starch and lipids are heated in a water bath to form a starch–lipid mixture. In a computer preset model, the starch–lipid mixture is placed into a food 3D printer, extruded through a nozzle, and freeze-dried to obtain RS.

For 3D printing, it is necessary to choose suitable resistant starch raw materials, such as high amylose corn starch, etc., that have high resistant starch content and specific physical and chemical properties and can show good formability and stability in the 3D printing process [102], and it is also necessary to add some auxiliary materials such as plasticizer glycerin, which can improve the flexibility and fluidity of starch, making it easier to extrude and shape in the printer [103]. Then, the resistant starch is thoroughly mixed with the auxiliary material. This can usually be achieved by mechanical stirring or high-speed mixing, as shown in Figure 3; for example, heat treatment can change the internal structure of starch granules and enhance their mechanical strength; enzymatic hydrolysis can adjust the length of the starch molecular chain and improve fluidity. After the mixed material is placed in the print cylinder, it needs to be properly pretreated, and the heating temperature can be controlled at 60–80 °C for 10–30 min [104]. The parameter settings of the printer mainly include extrusion temperature and printing speed. Depending on the properties of the resistant starch and auxiliary materials, the extrusion temperature is usually set between 60 and 180 °C. Higher temperatures help with the melting and extrusion of starch, but too high temperatures may cause starch degradation and affect product quality [105]. The printing speed is generally adjusted in the range of 10–50 mm/sec. A slower printing speed can ensure printing accuracy, but it will increase the printing time; faster printing speeds may lead to a decrease in molding quality, which needs to be optimized according to the specific printing model and requirements [106].

The results show that the formation of the ordered structure and V-shaped crystal structure significantly reduces the digestibility of starch, and catechins could loosely attach to starch chains, thereby facilitating binding to Trp59 of pancreatic α-amylase and preventing starch from binding to its active pocket [107]. Despite significant progress, there are still several challenges to achieving large-scale commercial production. The first is to ensure that stable rheological properties are maintained during the printing process, which is essential for achieving a high-quality product. The second is to address the compatibility between different types of starch, as each starch has unique physicochemical properties that may require individualized pretreatment steps [101].

### 3.5. Cold Plasma

Cold plasma is a partially ionized gas that contains ions, electrons, free radicals, and neutral particles, among other things. During the treatment of starch with cold plasma, these reactive particles interact with starch molecules [108].

Specialized cold plasma generators such as radio frequency (RF) or dielectric barrier discharge (DBD) plasma devices are often used. During the treatment process, appropriate treatment parameters need to be controlled, including the plasma power, treatment time, and gas atmosphere [109]. Cold plasma is capable of altering the microstructure and surface properties of starch granules through a variety of mechanisms. It can produce high-energy electrons at low temperatures, etc., and these components react with starch molecules, causing the starch chain to break, thus changing the molecular weight of starch. Secondly, cold plasma can induce an etching effect on the surface of starch granules, making starch granules more hydrophilic, which helps to improve their solubility and promote subsequent physical or chemical changes [110]. For example, the modification of glutinous rice, corn, and potato using carbon dioxide–argon radiofrequency-cooled plasma led to a significant increase in the gelatinization enthalpy and resistant starch content of the three waxy starches [111]. At the same time, cold plasma is often combined with other technologies. For example, the combination of cold plasma and sodium periodate to prepare dialdehyde starch caused the plasma treatment to break the starch molecular chain, resulting in a decrease in the viscosity of starch, increasing the contact area between starch and the oxidant sodium periodate, and increasing the aldehyde content by 9.98% compared with the traditional sodium periodate preparation method [112].

### 3.6. Ohmic Heating

Ohmic heating is based on the Joule heating effect that occurs when an electric current passes through the material. When an electric current passes through the starch suspension or starch gel, due to the difference in resistance between the starch granules and the surrounding medium, the electrical energy is converted into heat energy, which makes the inside of the material heat up rapidly [53]. In this process, a series of physical and chemical changes will occur in starch molecules, such as the expansion and gelatinization of starch granules and the rearrangement and cross-linking of molecular chains, thereby promoting the formation of resistant starch [113].

Compared with the traditional heating method, ohmic heating has the advantages of fast heating speed and uniform heating of materials. It has been found that the stability of starch gel is not affected by ohmic or conventional heating, which proves that ohmic heating can replace traditional heating without changing product characteristics [114].

### 3.7. Supercritical Fluids

In the process of preparing resistant starch, supercritical fluid (usually supercritical carbon dioxide) can penetrate into the starch granules, destroy the crystalline structure of starch, and rearrange and cross-link the starch molecular chain, thus forming resistant starch. At the same time, the special properties of supercritical fluids can promote the interaction of starch with other additives (such as enzymes, chemical reagents, etc.) and further change the structure and properties of starch [115].

The pretreated starch or starch–additive mixture is placed in an autoclave, sealed, and injected with supercritical carbon dioxide. By adjusting the temperature and pressure control system, the carbon dioxide reaches a supercritical state and is maintained at the set parameter conditions for treatment. For example, supercritical carbon dioxide treatment can increase the RS content and promote starch gelatinization; in addition, this treatment method is very effective in removing pesticides and microbial pollution [71].

The preparation of resistant starch using supercritical fluids has many advantages. For example, supercritical carbon dioxide is a non-toxic, odorless, non-flammable, and environmentally friendly solvent that does not produce harmful waste during treatment. Secondly, this method can realize the modification of starch under relatively mild conditions, which can reduce the destruction of starch nutrients and better maintain the natural characteristics of starch compared with traditional chemical modification methods. However, supercritical fluid is more often used as a green extraction method, and there is little research on the preparation of resistant starch; due to the high cost of equipment investment, professional operators are required to maintain and operate the equipment, which limits its application in small enterprises or laboratories to a certain extent. At the same time, the process parameters for the preparation of resistant starch using supercritical fluid need to be further studied and optimized so as to improve the yield and quality stability of resistant starch, reduce production costs, and make it more competitive for industrial applications.

## 4. Functions of RS

### 4.1. RS and Blood Glucose Control

The incorporation of RS exhibits remarkable advantages in blood glucose control for diabetic patients. Extensive research has revealed that, in comparison with RDS, RS offers superior regulation of fasting blood glucose levels, thereby highlighting its positive impact on enhancing blood glucose management [116]. The fermentation of RS by colonic bacteria results in the release of gases such as carbon dioxide, methane, and hydrogen, along with metabolically active SCFAs. These metabolites exert an influence on hepatic gluconeogenesis and insulin secretion, further contributing to the regulation of blood glucose [117]. An experiment was carried out on individuals who lead sedentary lifestyles and suffer from abdominal obesity. These participants were administered 75 g of digestible carbohydrates, along with muffins enriched with 30 g of RS. As a control, a 75-g glucose solution was orally administered. The results indicated that, two hours post-meal, the blood glucose levels of those who consumed the muffins fortified with 30 g of RS were significantly lower compared to the control group (12.5 ± 1.6 mmol/L·h vs. 15.6 ± 3 mmol/L·h, *p* = 0.002). Furthermore, the insulin levels were also markedly lower in comparison to the control group (1354.5 ± 6 pmol/L·h versus 1788.9 ± 522.8 pmol/L·h, *p* < 0.001). Upon further analysis, it was determined that the glycemic index of the muffin containing 30 g of RS was 48 [118].

RS can improve insulin sensitivity in patients. A comparison of 20 insulin-resistant subjects who consumed 40 g of RS per day versus those who did not consume RS showed that while RS did not significantly affect body weight, fat storage, or liver or visceral metabolism, it did improve insulin sensitivity in the RS-consuming group (insulin sensitivity as measured using the glucose clamp technique improved by 19% and worsened by 14% without RS compared to no RS) [119]. RS is generally thought to improve insulin sensitivity primarily by modulating the gut microbiota and promoting short-chain fatty acids, but in studies of mice with and without microbiota (mice fed a low-fat diet, a normal diet, and a normal diet supplemented with 10% RS2 or RS4), RS did not have a significant effect on body weight and energy intake; adipose tissue, macrophages, gut hormones, and adipokines were significantly affected, but RS also improved insulin levels in mice (RS2 and RS4 reduced insulin resistance by 83% and 45%, respectively), suggesting that RS improved insulin sensitivity in part independently of the microbiota [120].

RS can reduce total cholesterol and triglyceride levels and improve cecal mass, cecal wall mass, and wall surface area in mice, which are crucial for maintaining cardiovascular health [121]. The RS of lotus seed significantly lowers blood glucose levels and restores serum insulin levels, effectively regulating lipid metabolism disorders [122].

### 4.2. Resistant Starch and Colorectal Cancer Prevention

Colorectal cancer is one of the most common malignancies globally, and it has risen to be the third most prevalent cancer worldwide [123]. RS mainly prevents colorectal cancer by increasing the production of SCFAs and improving the intestinal microenvironment. RS generates SCFAs such as acetate and butyrate in the intestine. Butyrate serves as the primary energy source for colonic cells and exhibits antioxidant and anti-inflammatory properties, as well as the ability to induce apoptosis in tumor cells. Studies have shown that while the concentrations of acetate and propionate do not significantly inhibit the growth of human colorectal cancer (HCT-116) cells, reducing the levels of lactate and butyrate through in vitro fermentation enhances anti-colon cancer activity [124]. Overactive WNT signaling is often observed in colorectal cancer. Butyrate has been found to regulate the expression of WNT pathway components in multiple studies, such as inducing apoptosis in colon cancer cells, increasing the formation of β-catenin–T cytokine complexes, and regulating glycogen synthase kinase 3β activity, with different effects on different cell lines and genes [125]. One study demonstrated that RS affects secreted frizzle-associated protein 1 (SFRP1) and can lead to increased activity in the WNT pathway [126].

In terms of colorectal cancer prevention, evidence suggests that diets rich in red meat increase the risk of colorectal cancer, while consuming RS can reduce this risk [127]. RS can lower the concentration of secondary bile acids and the proliferation rate of colonic mucosal cells in the feces of healthy volunteers, which may decrease the risk of colorectal cancer [128]. A comparison between high-RS and low-RS diets revealed a 30% reduction in total neutral sterol concentrations in feces, with significantly lower fecal concentrations of total bile acids and secondary bile acids in the high-RS diet group compared to the low-RS diet group. These changes indicate that RS may play a role in cancer prevention by influencing bacterial metabolism in the human colon [129].

For individuals with Lynch syndrome (hereditary non-polyposis colorectal cancer), although supplementation with RS did not reduce the risk of developing colorectal cancer, it significantly decreased the incidence of other upper gastrointestinal cancers, such as pancreatic cancer and gastric cancer. A study involving nearly 1000 high-risk individuals showed that daily supplementation with 30 g of RS reduced the incidence of Lynch syndrome-associated tumors, particularly upper gastrointestinal cancers [130].

### 4.3. Impact on Gut Microbiota

**Figure 4:**
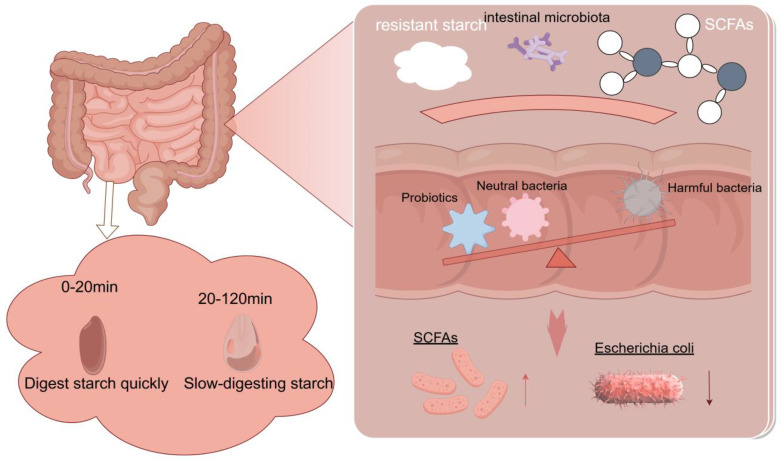
Schematic diagram of the intestinal fermentation of RS. RS cannot be enzymatically hydrolyzed in the small intestine and will enter the colon intact, producing short-chain fatty acids (SCFAs) through microbial action, increasing the number of beneficial bacteria in the intestine.

RS is fermented by gut microbiota in the large intestine, producing SCFAs such as acetate, propionate, and butyrate, among which butyrate plays a crucial role in improving gut health. A schematic diagram of resistant starch fermentation in the gut is shown in Figure 4. Butyrate is the most abundant SCFA produced during the digestion of RS, and it promotes the proliferation of normal colonic cells while inhibiting the proliferation of cancer cells [131]. SCFAs exert various effects on the human body, including lowering blood cholesterol and triglyceride levels, providing energy for colonic cells, and maintaining an appropriate colonic epithelial state [132]. Studies have shown that SCFAs serve as a link between gut microbiota and RS, as RS can improve the metabolism of gut microbiota, increase the abundance of beneficial microorganisms in the intestine, and reduce the abundance of harmful microorganisms such as *Escherichia coli* [133].

The impact of RS on the gut microbiome is diverse. Studies have demonstrated that mice fed with lotus seed RS3 exhibited an increased abundance of *Lactobacillus*, *Bifidobacterium*, *Lachnospiraceae*, *Ruminococcaceae*, and *Clostridium* species. These bacteria primarily influence starch digestibility and the efficiency of butyrate production [134]. Different thermal treatments (boiling, baking, and frying) of rice starch–rice protein–soybean oil ternary mixtures result in the destruction of starch crystal structure and intermolecular hydrogen bonds, leading to decreased acetate production and reduced SCFA content [135]. Furthermore, different types of RS have distinct effects on the gut. Research has shown that SCFA production from the simulated digestion of RS2 in high-amylose corn starch is significantly higher than that of RS3, RS4, and RS5. RS3 promotes the proliferation of *Bifidobacterium* primarily in the early stages of fermentation, while RS2 and RS4 show superior proliferation effects on *Bifidobacterium* in the later stages compared to RS3. RS2, RS3, and RS5 exhibit more pronounced proliferation effects on *Lactobacillus* in the later stages of fermentation than RS4 [28]. Different structural forms of RS, such as RS3, significantly promote the abundance of *Lactobacillus* in the gut. OS-starch (prepared using octenyl succinate anhydride) and cross-linked starch (prepared using sodium tripolyphosphate) significantly increase the abundance of *Lachnospiraceae* and *Enterococcus* in the gut, respectively. Therefore, the morphological structure of RS has a significant impact on microbial fermentation [136].

## 5. Applications of RS in Food

### 5.1. RS as a Food Additive

RS, as a novel food ingredient, has minimal impact on the sensory experience and flavor of food. RS starch can be chemically modified (otherwise, RS is just a food ingredient) as a food additive to improve nutrients that are lacking in certain foods. [137]. Transforming high-fat foods into low-fat alternatives is a significant challenge limited by cost, but RS can effectively control costs and enhance food quality as a fat substitute. Studies have found that adding modified starch to bread production improves bread characteristics [138]. Incorporating RS into mayonnaise increases its final viscosity and gel hardness, enhancing the stability of the mayonnaise [139]. By modifying starch into V-type complexes with fats, it can be used as a fat substitute to prepare low-fat meat products [140]. In some food processing applications, RS can also reduce the viscosity of batter, improve the texture and flavor of the finished product, and decrease moisture loss, thereby extending the shelf life of food and enhancing its nutritional value [141].

### 5.2. RS as Dietary Fiber

Dietary fiber refers to edible plant-based components, carbohydrates, and similar substances that are not digested and absorbed in the small intestine of humans, reaching the colon intact, being fermented by microbiota, and not providing calories from the diet [142]. RS fits this definition as it is not easily digested and absorbed in the small intestine but can enter the colon to be fermented by gut microbiota, producing beneficial short-chain fatty acids [143]. As a novel type of dietary fiber, RS has high dietary fiber content. Adding RS to food can significantly increase the dietary fiber component of the food. For instance, adding RS to chocolate reduces the total fat and protein content of the chocolate, increases its viscosity, decreases its hardness, and improves its quality as the RS content increases [144].

### 5.3. Resistant Starch as a Prebiotic

Prebiotics are dietary ingredients that are not digested and absorbed by the human digestive system but are selectively fermented, altering the composition or activity of the gut microbiota [145]. RS cannot be enzymatically hydrolyzed in the small intestine; thus, it is not digested and absorbed but instead passes intact into the colon, serving as a food source for probiotics in the colon and promoting their growth and reproduction [146]. Studies have found that lotus seed RS exhibits excellent prebiotic activity towards *Bifidobacterium adolescentis* and *Lactobacillus acidophilus* when cultured in a medium [147]. In an evaluation of the prebiotic properties of green banana RS, its specific growth rates for *Bacillus coagulans*, *Lactobacillus rhamnosus*, and *Saccharomyces boulardii* were relatively high, with short doubling times, revealing its potential as a prebiotic [148]. RS, as a prebiotic, promotes intestinal motility. Research has shown that buckwheat RS can enhance intestinal motility and has a laxative effect [149]. In the production of bread, adding RS significantly improved the fermentation time, hardness, and moisture content of the bread and affected the overall quality and sensory characteristics of the bread, and the total sensory (appearance, texture, flavor, etc.) of the bread was the highest in the sensory evaluation (RS, inulin, and polyglucose) [150].

### 5.4. Resistant Starch Facilitates Mineral Absorption

The SCFAs produced through the fermentation of RS in the intestine can lower the intestinal pH, promoting the dissolution and absorption of mineral elements such as calcium and magnesium, thereby enhancing the nutritional value of food [134]. Studies have indicated that RS may have a positive effect on intestinal mineral absorption. Fecal analysis of mice showed decreased levels of magnesium, calcium, iron, and zinc, suggesting better absorption of these minerals in the intestine [151]. A comparison between mice fed a wheat bran diet with added RS and a diet without RS showed that mice fed RS exhibited significantly improved phosphorus absorption in the intestine, which had a significant impact on the balance of trace elements such as calcium, magnesium, and zinc [152]. Adding different levels of RS (15, 20, and 25%) to pasta (with 15% wheat bran as a comparison) resulted in an increase in the total dietary fiber content of pasta, and the bioavailability of Ca, Fe, and Zn in pasta supplemented with RS was significantly higher than that in pasta supplemented with wheat bran (calcium: 43.5 ± 1.27 vs. 27.2 ± 2.16%, iron: 10.0 ± 0.14 vs. 6.0 ± 0.12%, Zinc: 29.5 ± 0.29 vs. 10.3 ± 0.31%), suggesting that RS can be a favorable source for high-fiber pasta production [153].

## 6. Conclusions

The significant health benefits of RS in improving digestive system health, regulating blood glucose levels, enhancing insulin sensitivity, aiding in weight control, increasing satiety, and preventing colon cancer will further consolidate its leading position in the health food market. As research continues to deepen and the market expands, the demand for RS will continue to grow, and its application fields will further broaden. At the same time, it is important to acknowledge that traditional RS preparation methods face challenges such as long preparation times, high production costs, difficult system control, and environmental safety concerns. In terms of basic research, studies on the impact of extrusion on starch structure have mainly focused on basic physicochemical properties, and the analysis of starch fine structure is not yet specific enough. Additionally, there is a lack of detailed analysis and elaboration on the mechanisms through which different physicochemical properties affect starch digestibility. Research on the digestibility of post-cooking products is also insufficient. Currently, there is still limited knowledge about the functional genes involved in RS synthesis, and it is challenging to improve and cultivate high-RS crop varieties due to a lack of theoretical support. Therefore, in the future, research on RS should aim to uncover its application possibilities within the realms of medicine and food science. It is crucial to delve into the long-term dynamic interactions between RS and the human intestinal microbiota, exploring how RS influences gut barrier function and immune regulation. Furthermore, investigations should focus on the potential mechanisms by which RS can contribute to the prevention and treatment of chronic diseases to enhance its application value in health management and medical fields.

## Figures and Tables

**Table 1:** Sources, characteristics, and production of different types of RS.

Types	Sources	Characteristics	Production	References
RS1	It is commonly found in partially ground grains and legumes	Starch granules that are difficult to digest due to the barrier action of the cell wall or the sequestration of proteins	Mildly milled grains and legumes	[11]
RS2	Raw potatoes, green bananas, high-amylose corn starch, and raw peas	These are types of starch that are naturally resistant to digestion	Naturally present	[12]
RS3	Cooling starch-based foods, fried foods, etc.	Starch that crystallizes during gelatinization and cooling or storage and is difficult to digest.	Enzymatic modification, physical modification, etc.	[13]
RS4	Chemical modification	Starch that becomes resistant to enzymatic degradation due to changes in its molecular structure and the introduction of certain chemical functional groups	Enzymatic debranching, moist heat treatment, annealing, pressure cooking, microwave radiation, etc.	[14]
RS5	The complex formed between amylose and lipids under specific conditions	Long, unbranched starch chains combine with free fatty acids to form a digestion-resistant helical structure	Classic synthesis, enzymatic synthesis, microwave heating, extrusion, etc.	[15]

**Table 2:** RS prepared using different raw materials and processing methods.

Starch Sources	Preparation Methods	RS Types	RS Yield	References
Rice starch	Enzymatic hydrolysis Heat-moisture treatment	RS3 RS3	12.66% 23.2%	[49] [50]
High-amylose wheat starch	Baking	RS3	11.9%	[51]
Wheat starch	Extrusion	RS5	35.47%	[52]
Cornstarch	Ohmic heating	RS3	8.29%	[53]
Waxy cornstarch	Enzymolysis	RS3	70.7%	[54]
High-amylose cornstarch	Mechanical activation	RS3	53.75%	[55]
Buckwheat starch	Heat-moisture treatment	RS3	41%	[56]
Tapioca starch	Pulsed electric field-assisted esterification	RS4	47.39%	[57]
Sweet potato starch	Cooking	RS3	54.96%	[58]
Potato starch	Toughening treatment	RS3	27.09%	[59]
Fava bean starch	Enzymatic hydrolysis + Retrogradation	RS3	64.88%	[60]
Pea starch	High-pressure homogenization	RS5	42%	[61]
Banana starch	Enzymolysis	RS3	68.99%	[62]
Yam starch	Autoclave Cross-linking processing Heat-moisture treatment	RS3 RS4 RS3	35.20% 42.42% 46.34%	[63]
Lotus seed starch	Autoclave	RS5	63.85%	[64]
Pueraria lobata starch	Temperature cycle crystallization	RS3	78.8%	[65]
Coix starch	Esterify	RS4	66%	[66]
Chestnut starch	Extrusion Heat-moisture treatment	RS5 RS3	12.35% 41.22%	[67] [68]

## Data Availability

No new data were created or analyzed in this study. Data sharing is not applicable to this article.
